# Two new genera and two new species of Mantophasmatodea (Insecta, Polyneoptera) from Namibia

**DOI:** 10.3897/zookeys.166.1802

**Published:** 2012-01-20

**Authors:** Benjamin Wipfler, Hans Pohl, Reinhard Predel

**Affiliations:** 1 Entomology Group, Institut für Spezielle Zoologie and Evolutionsbiologie, Friedrich-Schiller-University Jena, Erbertstr. 1, 07743 Jena, Germany; 2 Zoologisches Institut, Universität zu Köln, Zülpicher Str. 47b, 50674, Köln, Germany

**Keywords:** Mantophasmatodea, Polyneoptera, Lower Neoptera, *Pachyphasma*, *Striatophasma*, Namibia, Taxonomy

## Abstract

Two new species and two new genera (*Pachyphasma*, *Striatophasma*) of Mantophasmatodea are described from Namibia. *Pachyphasma brandbergense* is endemic to the Brandberg massif; *Striatophasma* occupies an extensive area south of the region inhabited by *Mantophasma*. Phylogenetic analyses (see Predel et al. in press) suggest a sistergroup relationship of *Striatophasma* and the South African Austrophasmatidae.

## Introduction

**Figure 1.**
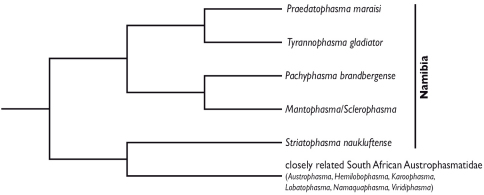
Simplified tree (midpoint-rooted) from a Bayesian phylogenetic analysis of peptide hormone sequences from southern African Mantophasmatodea (adapted from Predel et al. in press).

When Mantophasmatodea was described in 2002 (Klass et al. 2002), 88 years had passed since the last discovery of a new extant insect order (Grylloblattodea by Walker 1914). Hence the interest in this new taxon was tremendous. Within a few years studies on morphology, genetics, behaviour and peptides of Mantophasmatodea were published (e.g. Klass et al. 2003, Dallai et al. 2003, Predel et al. 2005, Beutel and Gorb 2008, Eberhard and Picker 2008). Simultaneously the number of described species as well as the geographic distribution increased: being originally described with one species from Namibia and one from southern Tanzania, a remarkable species diversity was found, particularly in South Africa (Picker et al. 2002, Klass et al. 2003, Eberhard and Picker 2008, Predel et al. in press). Up to now a total of 16 extant species in ten genera have been described (Klass et al. 2002, Klass et al. 2003, Zompro et al. 2003, Zompro and Adis 2006, Eberhard et al. 2011). The phylogenetic relationships within Mantophasmatodea as well as the taxonomic status of several genera have been subject of debate (Klass et al. 2003, Zompro et al. 2003, Zompro 2005, Zompro and Adis 2006, Damgaard et al. 2008, Eberhard et al. 2011) However, the separation and morphological description of species is sometimes difficult especially when based on few specimens. In a separate manuscript (Predel et al. in press) peptide mass fingerprints in combination with peptide sequencing were used to test the intraordinal phylogeny of Southern African Mantophasmatodea. Figure 1 shows a simplified version of the cladogram retrieved by Predel et al. (in press). Their analyses resulted in two monophyletic lineages: one containing all South African Austrophasmatidae (*Austrophasma* Klass et al., 2003, *Hemilobophasma* Klass et al., 2003, *Karoophasma* Klass et al., 2003, *Lobatophasma* Klass et al., 2003, *Namaquaphasma* Klass et al., 2003 and *Viridiphasma* Eberhard et al., 2011) and the Namibian genus *Striatophasma* gen. n. while the second clade contains all remaining Namibian species which are currently grouped in Mantophasmatidae. Within Mantophasmatidae *Praedatophasma* Zompro & Adis, 2002 + *Tyrannophasma* Zompro et al., 2003 and *Mantophasma* Zompro et al., 2002 + *Pachyphasma* gen. n. were retrieved as sistergroups. The present article describes these two new genera and species discovered by Predel et al. (in press).

## Methods

The terminology for the head and thorax follows Seifert (1995). The terms for abdominal structures are those used by Klass et al. (2003). The coloration refers to living specimens. Species descriptions are based on a designated holotype but all available specimens were taken into account in order to assess the intraspecific variation.

The information for the specimens is given in a standard manner, i.e., locality, geographic coordinates, elevation, date of collection (month indicated in lower case Roman numerals), habitat information, collector, depository, and preparation. Female (♀) and male (♂) symbols indicate the sex.

The following measurements are a selection from the proposed standards of Zompro et al. (2003) [abbreviations used by Zompro et al. (2003) in parentheses]: total length (a), length of pronotum (b), width of pronotum (c), length of mesonotum (b), width of mesonotum (c), length of metanotum (b), width of metanotum (c), length of the head (d), width of the head (f), head width over eyes (g), width between eyes (h), length of eye (i) and width of eye (j). Specimens were examined under a Zeiss Stemi SV11 with a calibrated ocular micrometer. A Leica MZ 12,5 with a camera lucida was used for line drawings.

A pair of each newly described species was dehydrated using increasing steps of ethanol up to 100%, dried at the critical point (Emitech K850 critical point dryer) and subsequently sputter-coated (Emitech K500). Scanning electron microscopy was performed on a Philips XL30 ESEM using a special specimen holder (Pohl 2010). Parts of each species are illustrated in the standard views of dorsal, lateral and ventral. The head is also in frontal view (frons being vertically) and the terminalia are in caudal view.

Peptide mass fingerprints and sequences were obtained by direct profiling of neuroendocrine tissues from single specimens using MALDI-TOF mass spectrometry as described in Predel et al. (in press).

The specimens referred below along with the abbreviations used in the text will be deposited in the following collections: NMNW – National Museum of Namibia, Windhoek, Namibia; ZFMK – Zoologisches Forschungsinstitut und Museum Alexander Koenig, Bonn, Germany; ZMUB – University of Bergen, Zoological Museum, Bergen, Norway.

## Taxonomy

### Striatophasma gen. n.

urn:lsid:zoobank.org:act:8B1618EC-5FBD-4C51-9301-7E0076EA742D

http://species-id.net/wiki/Striatophasma

#### Description and diagnosis.

*Striatophasma* gen. n. is placed as sistergroup to all remaining South African Austrophasmatidae sensu Klass et al. (2003) based on peptide hormone sequences (Predel et al. in press). Except for *Austrophasma gansbaaiense* and *Viridiphasma clanwilliamense* (Eberhard et al. 2011), it can be easily distinguished from the Austrophasmatidae sensu Klass et al. (2003) by its greenish colour. From all South African Austrophasmatidae, *Striatophasma* is separated by the lack of a butterfly-shaped dark median spot below the antennal base, genae that protrude from the compound eyes, the presence of spination in most body parts and its distribution in Namibia. From other Namibian mantophasmatodeans it can be distinguished by the presence of a brown dorso-median longitudinal stripe in males in combination with greenish body colouration. Asymmetric male genitalia and compound eyes that do not protrude from the genae distinguish *Striatophasma* from the East African *Tanzaniophasma*.

The new genus is characterized by a unique sequence of the adipokinetic hormone (pQVNF**T**P**S**Wamide; Predel et al. in press). The adipokinetic hormone sequence of all taxa currently grouped in Mantophasmatodae (*Mantophasma*, *Sclerophasma*, *Tyrannophasma*, *Praedatophasma*, *Pachyphasma*) is pQVNFSPGWamide (Gäde et al. 2005; Predel et al. 2011) and that of South African Austrophasmatidae is pQVNF**T**PGWamide (Predel et al. 2005).

#### Type species.

*Striatophasma naukluftense* sp. n.

#### Other included species.

None thus far.

#### Etymology.

The generic group name *Striatophasma* is a composition from the Latin word striatus meaning striped and the ending -phasma which is commonly used to term mantophasmatodeans. The gender is neuter.

### Striatophasma naukluftense sp. n.

urn:lsid:zoobank.org:act:C839900C-B908-4793-96E8-E9C80F06AA12

http://species-id.net/wiki/Striatophasma_naukluftense

Figs 2–10

#### Holotype.

Male, NAMIBIA: Naukluft Mountains, northern slope (Fig. 3), -24.15, 16.32, 1200–1400 m, 04.v.2006, R. Predel (NMNW), specimen in ethanol.

Paratypes, NAMIBIA: Naukluft Mountains: three males and four females; same data as holotype, one male and one female in ethanol (NMNW); one male and one female in ethanol (ZMUB); one female in ethanol and one male and one female critical point dried, sputter coated with gold (ZFMK). Nauchas: three males, -23.61, 16.37, 1750 m, 08.iv.2008, R. Predel (collection R. Predel), all in ethanol. Remhoogte: two males and two females, -23.96, 16.30, 1475 m, 08.iv.2008 R. Predel (collection R. Predel), specimen in ethanol.

#### Description male.

Measurements (male holotype followed by paratypes according to locality in parentheses, critical point dried specimen nor measured, in mm): total length (a): 27.1 (Naukluft mountains: 23.1, 27.3) (Nauchas: 23.6, 23.0, 22.3) Remhoogte (28.3, 25.6); length of pronotum (b): 4.0 (Naukluft: 4.1, 3.9) (Nauchas: 2.6, 2.9, 3.1) (Remhoogte: 3.6, 4.0); width of pronotum (c): 3.7 (Naukluft: 3.6, 3.0) (Nauchas: 3.3, 3.3, 3.4) (Remhoogte: 3.4, 3.7); length of mesonotum (b): 3.7 (Naukluft: 3.6, 3.7) (Nauchas: 3.4, 3.7, 3.7) (Remhoogte: 4.0, 4.1); width of mesonotum (c): 3.1 (Naukluft: 3.1, 3.0) (Nauchas: 2.9, 2.9, 3.0) (Remhoogte: 3.1, 3.3) ; length of metanotum (b): 3.2 (Naukluft: 2.4, 2.8) (Nauchas: 2.9, 2.9, 2.9) (Remhoogte: 3.1, 3.1); width of metanotum (c): 2.9 (Naukluft: 2.9, 2.9) (Nauchas: 2.7, 2.7, 2.9) (Remhoogte: 3.0, 3.1); length of the head (d): 3.9 (Naukluft: 3.3, 3.1) (Nauchas: 3.1, 3.3, 3.4) (Remhoogte: 3.9, 4.6); width of the head (f): 4.3 (Naukluft: 4.0, 3.9) (Nauchas: 3.7, 3.9, 4.0) (Remhoogte: 3.6, 3.7); head width over eyes (g): 4.6 (Naukluft: 4.4, 4.1) (Nauchas: 4.1, 4.1, 4.3) (Remhoogte: 4.0, 4.4); width between eyes (h): 2.7 (Naukluft: 2.6, 2.4) (Nauchas: 2.4, 2.6. 2.6) (Remhoogte: 2.9, 2.9); length of eye (i): 1.7 (Naukluft: 1.7, 1.7) (Nauchas: 1.7, 1.9, 1.9) (Remhoogte: 2.0, 2.1); width of eye (j): 1.0 (Naukluft: 0.9, 1.0) (Nauchas: 1.0, 1.0, 1.1) (Remhoogte: 1.1, 1.1).

Head (Fig. 4): globular, orthognathous, posteriorly covered by pronotum, bright green with brown dorsal stripe; compound eyes yellow-brownish with black spots; tip of lacinia and labial palps yellow. Head width similar to width of thorax, about as wide as long; sparsely covered with setae. Compound eyes tapered dorso-laterally and prominent, kidney-shaped, antero-ventral edge tapered, about twice as long as high; interoccular distance ca. the length of one eye, on vertex larger than on ventral eye margin; ocelli absent. Coronal and frontal suture indistinct, subgenal ridge well developed. Gena higher than compound eyes; ventral parts of occipital ridge very prominent; antennal sockets in between eyes, distinct; interantennal distance ca. diameter of one antennal socket; antennifer present; anterior tentorial pits dorsally of anterior mandibular articulation; frons with three bulges, one in between antennal sockets, two ventro-mesal of antennal sockets; frontoclypeal and temporal ridge not recognizable. Clypeus trapezoid, with well-developed clypeolabral ridge, four long setae in the dorsal clypeal area; oval sclerite in between clypeus and labrum present. Labrum flat, anteriorly rounded, with few short setae. Maxilla well developed, green with tip of lacinia yellow; maxillary palp yellow, five segmented, ca. 1.5 times longer than lacinia, covered with setae, palpomere one and two as long as wide, palpomere three 2.5 times as long as wide, palpomeres four and five ca. twice as long as wide. Labium green, tip of three segmented palp yellow. Scape and pedicel bright green. Scape as long as wide. Pedicle half as wide as scape, twice as long as wide dilating towards the tip. Flagellum yellow-greenish; ca. as long as the entire animal; 25 flagellomeres.

Thorax (Fig. 5): bright green, dorso-medially with longitudinal brown stripe that contains small green areas. Entire thorax covered with spines. Pronotum oval, scarcely covered with fine setae, with bulge positioned anterior-laterally; pronotum reachs over head and mesonotum. 2 cervicalia present, second postero-dorsally to first. Pleura subdivided into epimeron and episternum. Coxae large, covered with setae.

Legs: bright green, spikes in the tibial region black; covered with setae. Prothoracic leg more massive than meso- and metathoracic ones; femur three times as long as wide, with two ventro-median rows of green spikes. Tibia green, mesally whitish, ca. 6 times as long as wide, with two ventro-median rows of black spikes. Tarsus with five tarsomeres, first three green, fourth and fifth yellow, proximal four tarsomeres with euplantulae; arolium very large, yellow with black margin.

Wings: completely absent.

Abdomen: longer than thorax and head combined; bright green, meso-dorsal brown longitudinal stripe, enlarging posteriorly; populations of Naukluft mountains, Nauchas and Remhoogte differ in the shape of the stripe (Fig. 6). Tergites with lateral longitudinal brown stripe. Abdomen covered with setae. Abdominal tergum I same width as metathorax; terga slightly broadening towards tergum VIII, terga IX and X narrowing again. Pleura bright green. Sterna bright green with median brown stripe.

Male terminalia (Fig. 7): tergum IX bright green, mesal brown stripe; shorter than tergum VIII, postero-ventral border with dorsal bend in lateral view. Tergum X bright green, mesal brown stripe, roof-shaped in lateral view. Subgenital plate (sternite IX) green with brown-reddish areas; process of subgenital plate broad, dorsal part arch-shaped when seen from posterior, broadly emarginated dorsally. Cerci one segmented, base green, distally brown-reddish; densely covered with setae; diameter mesally round, uniformly curved, slightly narrowed towards the apex; dorsal projection of cercus very small, located directly dorsally to apex; cerci extending towards the middle of the subgenital plate.

**Description female.**For the female only differences to the male are described.

Measurements (critical point dried specimen not measured, in mm): total length (a): (Naukluft: 23.6, 29.0, 31.9) (Remhoogte: 32.0, 35.4); length of pronotum (b): (Naukluft: 4.0, 3.9, 4.3) (Remhoogte: 4.3, 4.4); width of pronotum (c): (Naukluft: 3.7, 3.7, 3.7) (Remhoogte: 4.1, 4.3); length of mesonotum (b): (Naukluft: 3.9, 3.4, 3.0) (Remhoogte: 4.6, 4.6); width of mesonotum (c): (Naukluft: 3.3, 3.3, 3.3) (Remhoogte: 3.9, 3.9); length of metanotum (b): (Naukluft: 3.1, 2.9, 3.3) (Remhoogte: 3.1, 3.7); width of metanotum (c): (Naukluft: 3.1, 3.1, 3.1) (Remhoogte: 3.7, 3.7); length of the head (d): (Naukluft: 4.4, 3.7, 3.7) (Remhoogte: 4.9, 5.7); width of the head (f): (Naukluft: 4.7, 4.4, 4.5) (Remhoogte: 4.6, 4.7); head width over eyes (g): (Naukluft: 4.9, 4.6, 4.7) (Remhoogte: 4.7, 5.3); width between eyes (h): (Naukluft: 3.1, 2.7, 3.0) (Remhoogte: 3.4, 3.7); length of eye (i): (Naukluft: 1.2, 1.1, 1.1) (Remhoogte: 1.3, 1.3); width of eye (j): (Naukluft: 2.1, 1.9, 2.0) (Remhoogte: 2.4, 2.7).

Head (Fig. 8): moss green, dorso-median brown stripe absent; labrum, tip of lacinia and labial palps yellow; compound eyes brownish with reddish stripe. Mouthparts similar to male. Compound eyes prominent, kidney-shaped, dorso-laterally positioned, smaller than in the male, twice as long as high. Interoccular distance ca. 1.5 times the diameter of one eye, on vertex smaller than on ventral eye margin. Temporal ridge distinct. Distance between antennal sockets ca. 1.5 times the diameter of one antennal socket.

Thorax (Fig. 9): moss green with dark green meso-dorsal stripe; thoracic nota with lateral brown dots.

Legs: reddish-green, with brown dots where setae emerge; covered with setae; two ventro-median rows of spikes present on tibia and femur; spikes on tibia black.

Abdomen: moss green, dark green meso-dorsal stripe representing dorsal vessel, lateral margins of terga whitish. Tergum I as broad as metanotum. Pleura and sternites moss green.

Female terminalia (Fig. 10): tergum IX moss green, half the length of tergum VIII.Tergum X moss green, twice as long as tergum IX; apex rounded posteriorly; epiproct moss green, short, twice as broad as long; paraprocts moss green, densely covered with setae. Cerci moss green, densely covered with setae, cone shaped, reaching the tips of the paraprocts. Sternite VIII moss green, acuminated posteromesally. Gonapophysis VIII long and slender, distally blunt with ventrocaudal process. Gonocoxite IX trapezoid in lateral view; gonoplac triangular, heavily sclerotised.

#### Etymology.

The species is named after the type locality, the Naukluft Mountains.

#### Comments.

Specimens were mainly found in dwarf shrubs. Adult specimens are obviously not associated with grass tussocks.

### Pachyphasma gen. n.

urn:lsid:zoobank.org:act:E43A1051-968A-4DFE-BB94-EDD0AB742B07

http://species-id.net/wiki/Pachyphasma

#### Description and diagnosis.

Based on peptide hormone sequences, *Pachyphasma* gen. n. is placed as sistergroup to *Mantophasma*/*Sclerophasma* (Predel et al. in press). The clade *Pachyphasma* gen. n. + *Mantophasma*/*Sclerophasma* was determined as sistergroup to *Tyrannophasma* + *Praedatophasma*. *Pachyphasma* gen. n. can be distinguished from all other mantophasmatodeans by an abdomen that is shorter than the thorax and a metanotum that is broader than the abdominal tergum I. *Pachyphasma* was found in the same biotope/habitat as *Tyrannophasma gladiator*, but on flowering bushes of Compositae while *Tyrannophasma* mainly inhabits grass tussocks but never these bushes.

The new genus is characterized by several distinct neuropeptide sequences, e.g. periviscerokinin-1 (EAAGLIAFPRTamide) (Predel et al. in press). The respective mass signal ([M+H]^+^: 1144.6) can be detected in preparations of abdominal perisympathetic organs of males/females/nymphs.

#### Type species.

*Pachyphasma brandbergense* sp. n.

#### Other included species.

None thus far.

#### Etymology.

the generic group name *Pachyphasma* is a composition from the Greek word pachys meaning thick and the ending -phasma which is commonly used to term mantophasmatodeans. The gender is neuter.

**Figure 2.**
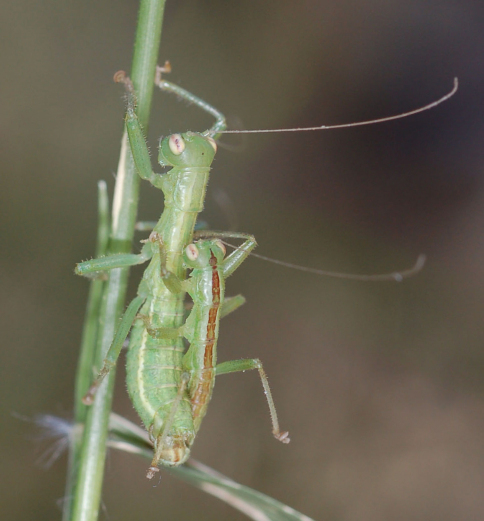
Habitus photography of *Striatophasma naukluftense* sp. n.; copula with smaller ♂ on top of ♀.

**Figure 3.**
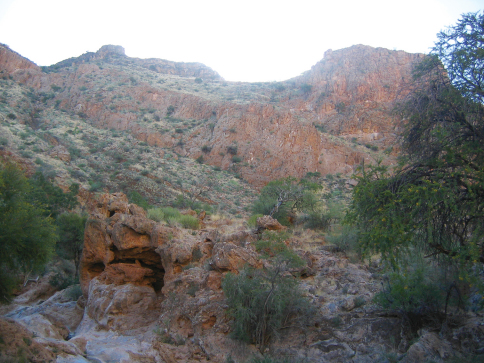
Type locality of *Striatophasma naukluftense* sp. n, Naukluft Mountains, Namibia.

**Figure 4.**
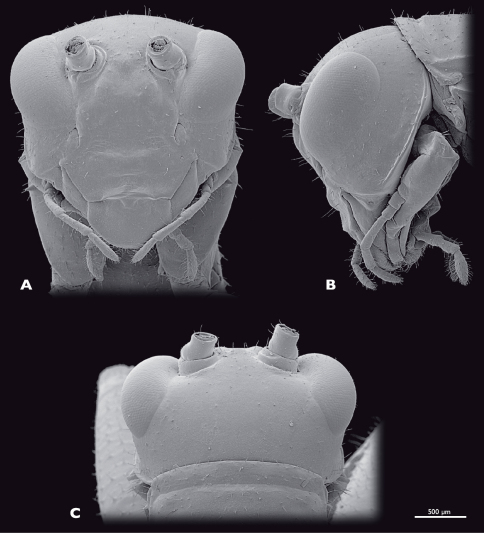
Head of ♂ *Striatophasma naukluftense* sp. n., SEM- micrographs **A** frontal view **B** lateral view **C** dorsal view.

**Figure 5.**
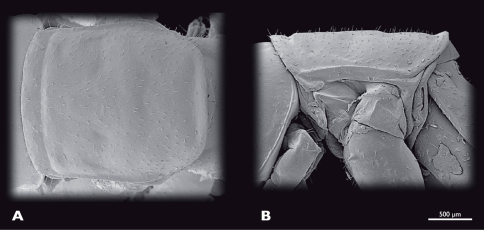
Prothorax of ♂ *Striatophasma naukluftense* sp. n., SEM- micrographs **A** dorsal view **B** lateral view.

**Figure 6.**
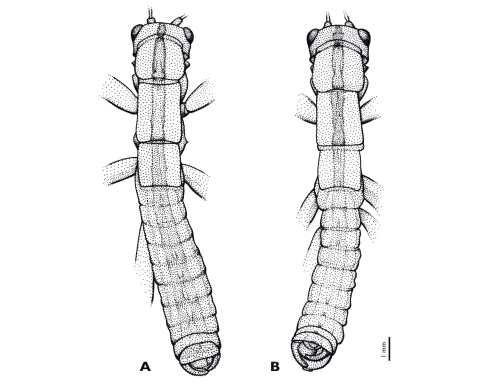
Variations of brown longitudinal stripe in *Striatophasma naukluftense* sp. n. **A** Holotype, male from Naukluft mountains **B** Paratype, male from Nauchas.

**Figure 7.**
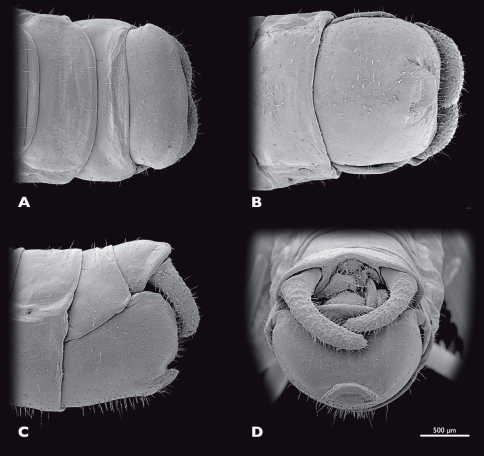
Terminalia of ♂ *Striatophasma naukluftense* sp. n., SEM- micrographs **A** dorsal view **B** ventral view **C** lateral view **D** caudal view.

**Figure 8.**
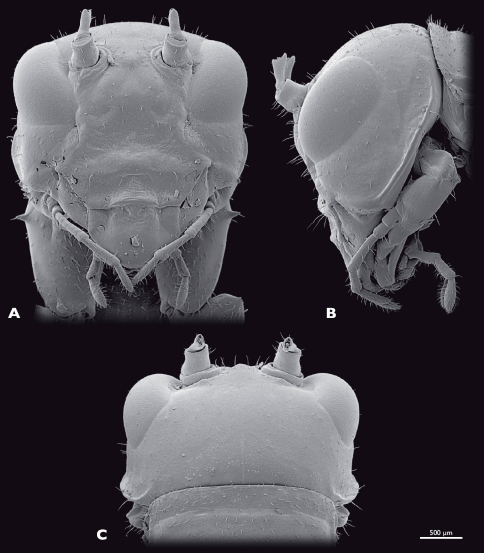
Head of ♀ *Striatophasma naukluftense* sp. n., SEM- micrographs **A** frontal view **B** lateral view **C** dorsal view.

**Figure 9.**
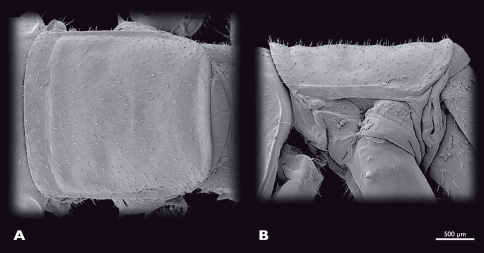
Prothorax of ♀ *Striatophasma naukluftense* sp. n., SEM- micrographs **A** dorsal view **B** lateral view.

**Figure 10.**
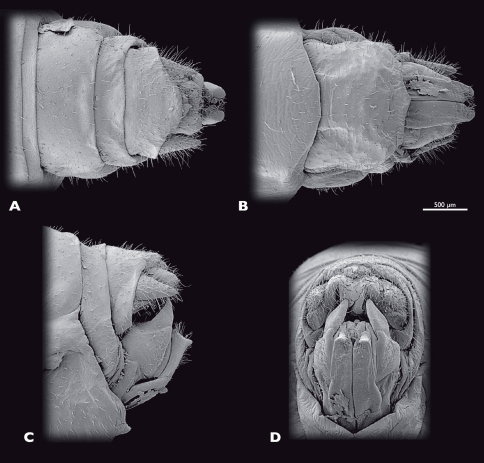
Terminalia of ♀ *Striatophasma naukluftense* sp. n., SEM- micrographs **A** dorsal view **B** ventral view **C** lateral view **D** caudal view.

### Pachyphasma brandbergense sp. n.

urn:lsid:zoobank.org:act:A01E0850-5300-433B-A83A-A68C12C0755D

http://species-id.net/wiki/Pachyphasma_brandbergense

Figs 11–18

#### Holotype male.

NAMIBIA: Brandberg Plateau, south of Königstein, with sclerophyllous vegetation (Fig. 12), -21.16, 14.58, +/-2000 m, 26.iv. 2006, R. Predel. (NMNW), specimen in ethanol.

Paratypes NAMIBIA: Brandberg Plateau, south of Königstein: four males and eight females; same data as holotype; one male and two females in ethanol, one pair in copula in ethanol (NMNW); one male and two females in ethanol (ZMUB); two females in ethanol and one male and one female critical point dried, sputter coated with gold (ZFMK). Two juveniles and six heavily damaged specimens (1 juvenil, 2♀, 3♂) were excluded from the type-series (collection R. Predel).

#### Description male.

Measurements (male holotype followed by paratypes in parentheses, critical point dried male and male in copula not measured, in mm): total length (a): 23.0 (18.1, 17.9); length of pronotum (b): 3.9 (2.9, 3.1); width of pronotum (c): 3.0 (3.0, 3.4); length of mesonotum (b): 3.6 (2.7, 2.9); width of mesonotum (c): 2.9 (2.7, 3.4); length of metanotum (b): 3.0 (2.7, 2.3); width of metanotum (c): 2.7 (2.4, 2.6); length of the head (d): 4.4 (4.0, 3.9); width of the head (f): 4.0 (3.7, 3.9); head width over eyes (g): 4.4 (4.3, 4.3); width between eyes (h): 3.0 (2.7, 2.6); length of eye (i): 2.0 (2.0, 2.1); width of eye (j): 1.0 (1.0, 1.0).

Head (Fig. 13): nearly triangular when seen frontally, orthognathous. Bright green; thin dorso-median brown longitudinal stripe; broader lateral brown longitudinal stripes posterior of the compound eyes; vertex, frons and subgena with reddish areas; head capsule with brown spots where setae emerge; compound eyes grey with reddish stripe; mouthparts including palpi and labrum greenish. Head capsule slightly broader than thorax; sparsely covered with setae. Compound eyes kidney-shaped, prominent, globular, about 1.5 times as long as high; interoccular distance ca. the length of eye, on vertex larger than on ventral eye margin; ocelli absent. Coronal and frontal suture as well as frontoclypeal and temporal ridge indistinct; subgenal ridge distinct, with bend ventrally of compound eye; distance between antennal sockets same as diameter of one socket; anterior tentorial pits dorsomesally of anterior articulation of mandible. Frons with three bulges, two ventromedial of antennal sockets, one in between the antennal sockets. Clypeus trapezoid, with four long setae dorsally; oval sclerite in between clypeus and labrum. Labrum greenish, anteriorly rounded, flat sparsely covered with setae. Maxilla of orthopteroid type, sparsely covered with setae; maxillary palp five-segmented, green, palpomeres two to five covered with setae, palpomere one and two short, nearly as wide as long, palpomere three 2.5 times longer than wide, palpomere four and five ca. twice as long as wide. Labial palp three segmented, green. Scape and pedicel bright green. Scape conical, at base as long as wide. Pedicle half as wide as scape, twice as long as wide. Flagellum yellow-greenish; nearly as long as the entire animal; 25 flagellomeres.

Thorax (Fig. 14): compact. Bright green; thin meso-dorsal brown stripe that ends in the anterior third of metanotum; lateral edges of all three nota with thick brown stripe; lateral margin of nota white; pleurae bright green with reddish areas. Completely covered with setae; brown spots where setae emerge along entire thorax. Pronotum nearly squared; overlapping head and mesonotum. Mesonotum not as wide as pronotum, nearly squared. Metanotum narrower than mesonotum; narrowing posteriorly. Coxae large.

Legs: bright green. Completely covered with setae; brown spots where setae emerge. Prothoracic leg more massive than meso- and metathoracic leg. Tarsus with 5 brownish tarsomeres; proximal four with euplantulae; large arolium present.

Wings: completely absent.

Abdomen: shorter than thorax: enlarging posteriorly; completely covered with setae. Bright green; lateral brown longitudinal stripes on tergites. Abdominal tergum I narrower than metanotum; terga slightly broadening towards tergum VIII; terga IX and X narrowing again. Pleura bright green with yellowish stripe. Sterna bright green.

Male terminalia (Fig. 15): tergum IX bright green, lateral brown stripe; ca. same length as tergum VIII; postero-ventral border rounded. Tergum X bright green; lateral brown longitudinal stripes; roof-shaped in lateral view. Subgenital plate (sternite IX) green; broadly emarginated dorsally; process of subgenital plate broad; dorsal part horizontal in posterior view. Cerci one segmented; green; densely covered with setae; diameter mesally oval; uniformly curved; dorsal projection of cercus very prominent and located at two-third of length of cercus; cerci extending towards middle of subgenital plate.

**Description female.**For the female only differences to the male are described.

Measurements (critical point dried female and female in copula not measured, in mm):

Total length (a): 20.1, 17.4, 28.3, 27.8, 15.9, 20.3; length of pronotum (b): 4.0, 4.1, 4.0, 3.9, 3.6, 3.7; width of pronotum (c): 4.3, 4.3, 3.8, 3.9, 4.1, 3.7; length of mesonotum (b): 3.1, 2.9, 3.6, 3.7, 2.9, 2.9; width of mesonotum (c): 3.7, 3.7, 3.4, 3.3, 3.7, 3.1; length of metanotum (b): 2.9, 2.2, 2.3, 2.3, 2.6, 2.4; width of metanotum (c): 3.6, 3.4, 3.7, 3.4, 3.6, 3.1; length of the head (d): 1.1, 1.2, 1.2, 1.1, 1.0, 1.1; width of the head (f): 5.2, 4.4, 4.4, 4.5, 4.4, 4.2; head width over eyes (g): 5.4, 5.1, 5.0, 4.9, 4.7, 4.6; width between eyes (h): 3.4, 2.9, 2.9, 3.0, 2.9, 2.9; length of eye (i): 2.5, 2.3, 2.3, 2.1, 2.3, 2.3; width of eye (j): 1.1, 1.2, 1.3, 1.1, 1.1, 1.1.

Head: (Fig. 16): globular. Bright green; thin dorso-median brown longitudinal stripe; broader lateral brown longitudinal stripes posterior of compound eyes. Compound eyes less bulged than in male. Scape and pedicel green; 25 flagellomeres.

Thorax (Fig. 17): compact. Bright green; thin meso-dorsal brown longitudinal stripe; thick lateral brown longitudinal stripe on notae; lateral margin of nota white; pleura bright green.

Abdomen: bright green, dark green meso-dorsal longitudinal stripe; pleura bright green with yellowish longitudinal stripe; sternites bright green.

Female terminalia (Fig. 18): tergum IX bright green. Tergum X moss green; 1.5 times as long as tergum IX; apex rounded posteriorly. Paraprocts bright green; sparsely covered with setae. Cerci bright green; densely covered with setae; triangular; protruding further caudally than paraprocts. Sternite VIII bright green; posterior margin rounded. Gonapophysis VIII long and slenderd distally blunt; with very short ventrocaudal process. Gonocoxite IX trapezoid in lateral view. Gonoplac triangular; heavily sclerotised.

#### Etymology.

The species is named after the type locality, the Brandberg massif.

**Figure 11.**
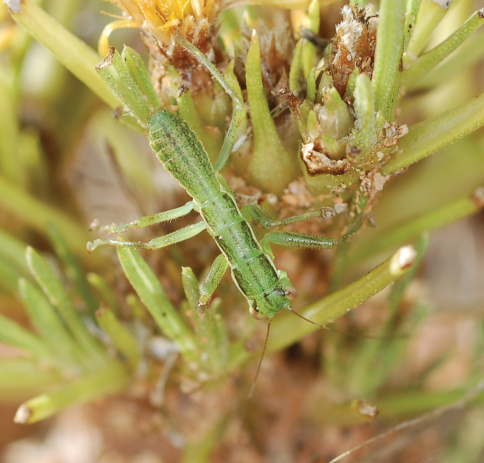
Habitus photography of ♀ *Pachyphasma brandbergense* sp. n.

**Figure 12.**
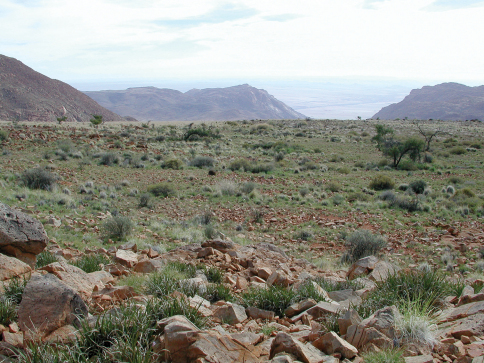
Type locality of *Pachyphasma brandbergense*sp. n, Brandberg Plateau, Namibia.

**Figure 13.**
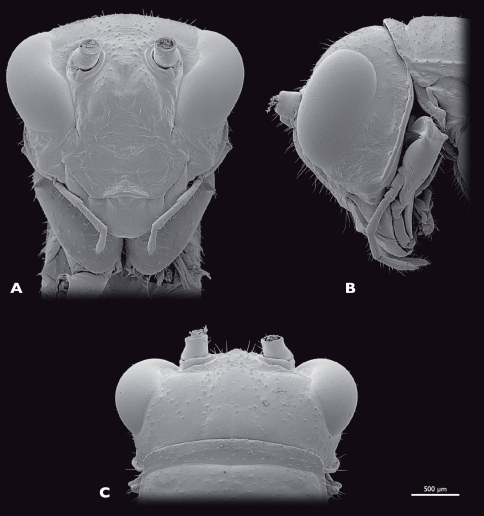
Head of ♂ *Pachyphasma brandbergense*sp. n., SEM- micrographs **A** frontal view **B** lateral view **C** dorsal view.

**Figure 14.**
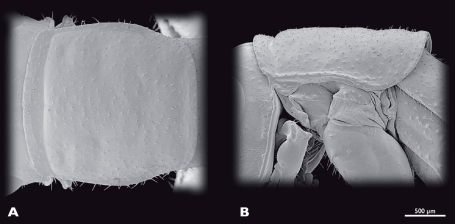
Prothorax of ♂ *Pachyphasma brandbergense*sp. n., SEM- micrographs **A** dorsal view **B** lateral view.

**Figure 15.**
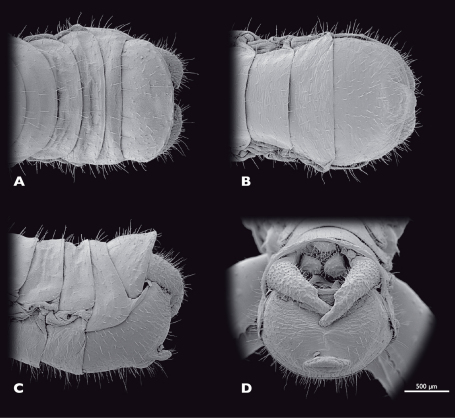
Terminalia of ♂ *Pachyphasma brandbergense*sp. n., SEM- micrographs **A** dorsal view **B** ventral view **C** lateral view **D** caudal view.

**Figure 16.**
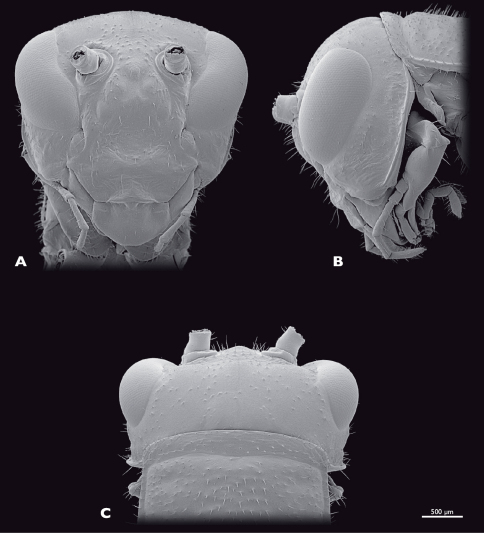
Head of ♀ *Pachyphasma brandbergense*sp. n., SEM- micrographs **A** frontal view **B** lateral view **C** dorsal view.

**Figure 17.**
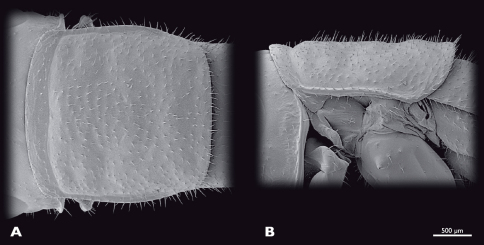
Thorax of ♀ *Pachyphasma brandbergense*sp. n., SEM- micrographs **A** dorsal view **B** lateral view.

**Figure 18.**
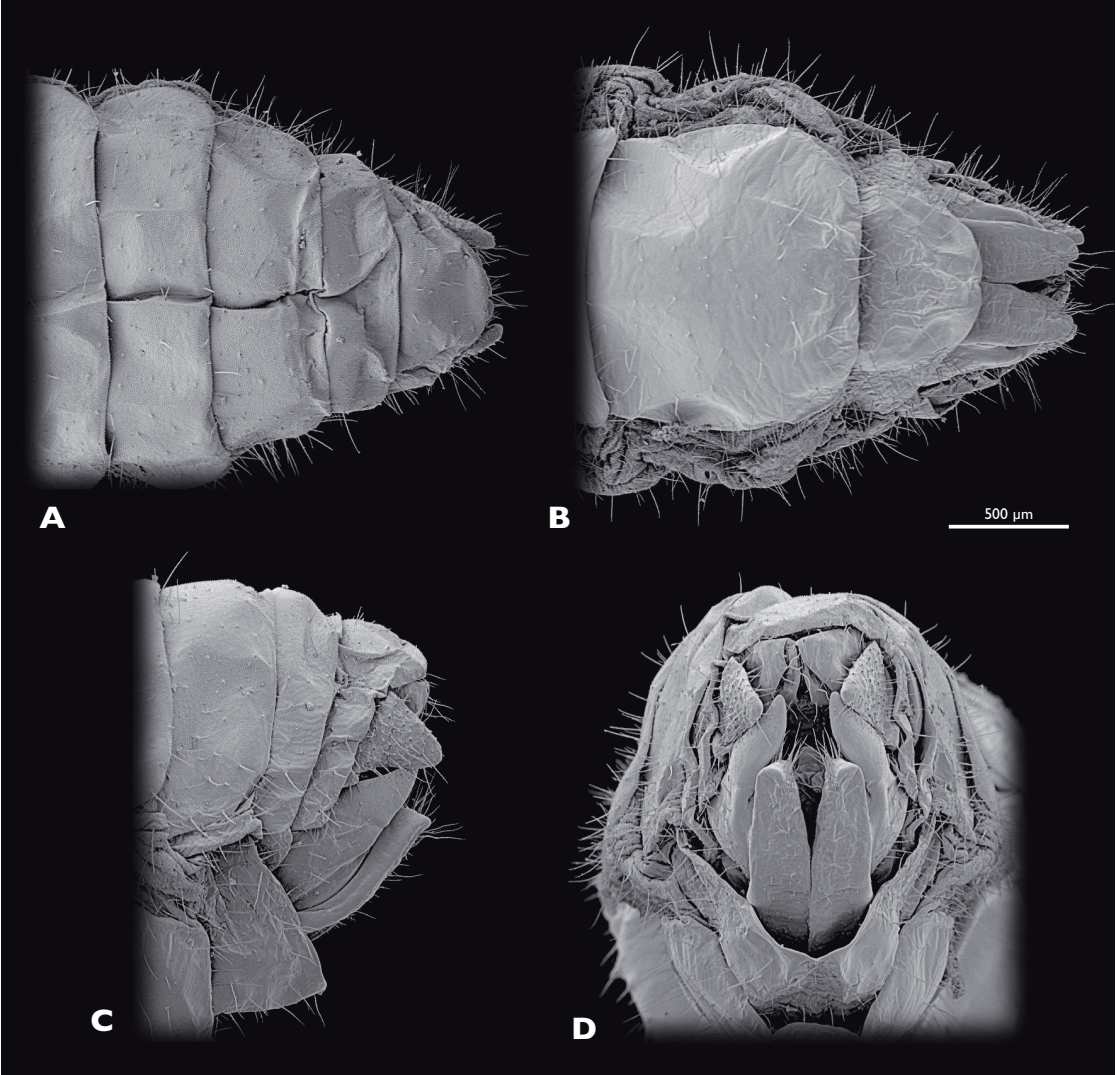
Terminalia of ♀ *Pachyphasma brandbergense*sp. n., SEM- micrographs **A** dorsal view **B** ventral view **C** lateral view **D** caudal view.

## Discussion

**Figure 19.**
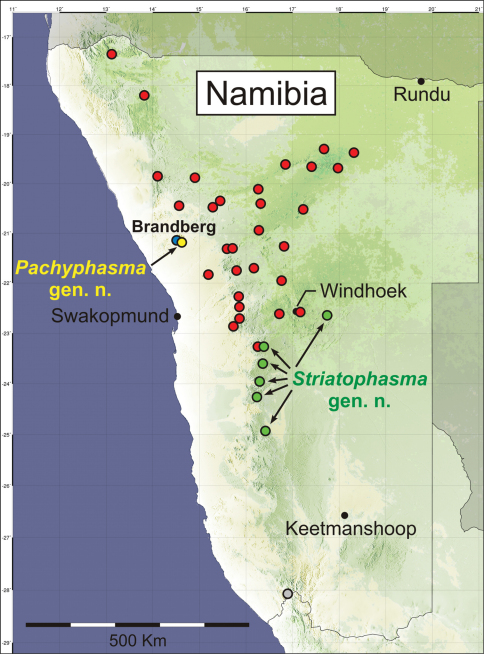
Collection localities of Mantophasmatodea from Namibia. Red, *Mantophasma* (incl. *Sclerophasma paresisense*); green, *Striatophasma* gen. n.; blue, *Tyrannophasma*; yellow, *Pachyphasma* gen. n.; gray, *Praedatophasma* (Predel et al. in press).

The discovery of the two new genera of Mantophasmatodea results from extensive collecting activities during the last years (Fig. 19). With it, it can be expected that, at least in Namibia, the majority of higher taxa are now known. It is particularly remarkable, that within the large distributional range of *Mantophasma*, additional mantophasmatodean taxa have not been found (see Predel et al. in press). *Pachyphasma brandbergense* may represent a phylogenetic relict which survived only on the Brandberg Massif, an isolated biotope outside the currently accessible range of *Mantophasma*. At the plateau of the Brandberg massif, *Pachyphasma brandbergense* occurs sympatrically with the much larger *Tyrannophasma gladiator*. Nevertheless, both species occupy different ecological niches.

In contrast to *Pachyphasma* gen. n., the newly described *Striatophasma* gen. n. represents a widely distributed taxon. Its northernmost known distributional limit seems to be equivalent to the southern limit of *Mantophasma* (Predel et al. in press). The general distribution of these taxa suggests that *Striatophasma* is better adapted to conditions of lower rainfall and scattered vegetation. In the field, different morphotypes from different localities of *Striatophasma* were found with a variable development of the brown dorsal stripe in males (Fig. 6).

Thus far, the monophyly of *Pachyphasma* and *Striatophasma* is supported by distinct morphological features and a number of specific peptide hormone sequences which clearly separate these taxa from all other (Predel et al. in press). Most peptide hormones are synthesized in the central nervous system and are released via neurohemal organs which accumulate these messenger molecules. In insects, the ganglia of the different tagmata (head, thorax, abdomen) have each their specific external release sites with tagma-specific peptide hormones which can be analysed by mass spectrometry (see Predel et al. 2005). It is beyond the scope of this manuscript and also beyond our current state of knowledge to present a morphology-based diagnostic key for the separation of all mantophasmatodeans. More data, particularly about within-species variation, are required to address this problem. For the differentiation of the major lineages of Mantophasmatodea, however, mass fingerprints of peptide hormones provide unambiguous information (see Predel et al. 2005, in press) which can be used in a dichotomic key. This character set is already studied in detail with respect to Mantophasmatodea and can be obtained from single specimens (males, females or larvae). It is thus possible to assign newly collected specimens to previously analyzed taxa simply and rapidly and we encourage entomologists to contact one of us in case of problems in obtaining such data. Alternatively, we offer to perform immediate and free of charge analyses for the collector if living or frozen specimens can be provided (we are also working on the development of methods to analyze insects preserved in ethanol).

### Dichotomic key based on peptide barcoding (peptide hormones; RC, retrocerebral complex; aPSO/tPSO, abdominal/thoracic perisympathetic organ):

**Table:** 

1	RC (952.4 + 1350.6)	Austrophasmatidae sensu Klass et al. 2003 (*Hemilobophasma*, *Austrophasma*, *Karoophasma*, *Namaquaphasma*, *Lobatophasma*, *Viridiphasma*)
–	RC without (952.4 and 1350.6)	2 (remaining Mantophasmatodea)
2	RC (982.4 + 1369.6)	*Striatophasma*
–	RC (938.4 + 1369.6)	3
3	aPSO (1144.7), tPSO (1066.5)	*Pachyphasma*
–	aPSO without (1144.7), tPSO without (1066.5)	4
4	aPSO (1735.8), tPSO (1174.6)	*Praedatophasma*
–	aPSO without (1735.8), tPSO without (1174.6)	5
5	aPSO (1772.8), tPSO (819.5)	*Tyrannophasma*
–	aPSO without (1742.8) tPSO without (819.5)* Mantophasma, Sclerophasma, Tanzaniophasma*

## Supplementary Material

XML Treatment for Striatophasma

XML Treatment for Striatophasma naukluftense

XML Treatment for Pachyphasma

XML Treatment for Pachyphasma brandbergense
